# Regional Gray Matter Volume Mediates the Relationship Between Conscientiousness and Expressive Suppression

**DOI:** 10.3389/fnhum.2018.00301

**Published:** 2018-08-14

**Authors:** Cheng Chen, Yu Mao, Jie Luo, Li He, Qiu Jiang

**Affiliations:** ^1^School of Psychology, Southwest University, Chongqing, China; ^2^Key Laboratory of Cognition and Personality, Ministry of Education, Chongqing, China; ^3^School of Chinese Language and Literature, Southwest University, Chongqing, China; ^4^School of Education, Chongqing Normal University, Chongqing, China

**Keywords:** conscientiousness, inferior frontal gyrus, emotion regulation, expressive suppression, cognitive reappraisal

## Abstract

Conscientiousness is frequently characterized by tendencies to be self-disciplined, to demonstrate organization and dependability, to act dutifully, to aim for achievement and to have good impulse control; this trait plays an important role in some special contexts, such as legal consciousness. Although a great number of studies have confirmed the impact of conscientiousness on emotion experience, little is known about the relationship between conscientiousness and emotion regulation, or of the brain structural basis that is involved. The current study investigated the neuroanatomical basis of the relationship between conscientiousness and emotion regulation from the perspective of individual differences. The voxel-based morphometry (VBM) method at the whole-brain level was used to identify the brain structural basis related to conscientiousness in a large, young sample (*n* = 351). The results showed that conscientiousness was significantly and positively correlated with the gray matter volume (GMV) in the right inferior frontal gyrus (IFG), which is the key region for inhibitory control. Further mediation analysis revealed that the IFG volume partially mediated the relation between conscientiousness and expressive suppression (ES), rather than cognitive reappraisal (CR), which showed that the IFG is associated with direct inhibitory control and plays a specific role in the relationship between conscientiousness and the two strategies of emotion regulation. Taken together, these findings contributed to sharpening the understanding of the correlation between conscientiousness and emotion regulation from the perspective of the brain structural basis.

## Introduction

In the Big Five taxonomy of personality, conscientiousness has often been defined as a personality trait that reflects the tendencies of self-control, hard work, achievement-striving and acting dutifully (McCrae and John, 1992; DeYoung et al., 2010; Roberts et al., 2014). Conscientiousness may play an important role in some special fields, such as legal consciousness (Hirsh and Lyons, 2010). Specifically, individuals with higher levels of legal consciousness always exhibit greater self-control (Chua, 2008; Goncharova, 2016), which is thought to be a core component of conscientiousness (Roberts et al., 2005, 2014) and also affects emotional experience (Gallagher, 2006). Meanwhile, recent studies revealed that conscientiousness is moderately related to both positive and negative emotion experiences (DeNeve and Cooper, 1998; Kotov et al., 2010; Fayard et al., 2012; Javaras et al., 2012). More specifically, meta-analyses found a strong positive correlation between conscientiousness and subjective well-being (DeNeve and Cooper, 1998); conversely, conscientiousness was negatively correlated with fear, guilt experience and general negative affect (Fayard et al., 2012). Conscientious people tend to be self-disciplined, efficient and dutiful and to aim for achievement, this pattern of behavior allows them to experience greater happiness and well-being (DeNeve and Cooper, 1998); people with low levels of conscientiousness always conduct more easy-going, disorganized and spontaneous lives, which may cause them to experience poor interpersonal relationships and failures at work and can more easily lead to negative affective consequences (Fayard et al., 2012). Although a great number of studies have confirmed the impact of conscientiousness on emotion experience, little is known about the relationship between conscientiousness and emotion regulation.

Previous studies have suggested that individuals with high conscientiousness may be better at emotion regulation and, particularly, that conscientiousness can predict greater recovery from negative emotion (Javaras et al., 2012). Although the definition of conscientiousness does not involve emotion regulation, self-control or self-regulation is considered the core component of conscientiousness (Caspi et al., 2005; Roberts et al., 2005, 2014; Eisenberg et al., 2014), which is closely related to emotion regulation (Etkin et al., 2015). Empirical observations have shown that the greater conscientiousness people report, the less anger they feel and conscientiousness was also found to moderate the relationship between anger and aggressive behavior in frustrating experiences (Jensen-Campbell et al., 2007). Furthermore, combined with other existing studies, it seems that there is a moderate link between conscientiousness and emotion regulation (Gross and John, 2003; Javaras et al., 2012), for example, a study indicated that emotion regulation ability is positively correlated with conscientiousness (Ivcevic and Brackett, 2014) and another study also found that conscientiousness could predict greater recovery from the negative emotional stimuli (Javaras et al., 2012). However, to our knowledge, no research has directly focused on the association between conscientiousness and emotion regulation, or on the neuroanatomical basis that is involved. Thus, in this study, we aimed to explore the potential relationship between conscientiousness and emotion regulation and to elucidate the brain structure between them in a large, young sample.

Emerging neuroimaging evidence has suggested that conscientiousness is closely related to the brain areas involved in the voluntary control of behavior. Structural magnetic resonance imaging (MRI) studies revealed a positive correlation between conscientiousness and gray matter volume (GMV) in the lateral prefrontal cortex (DeYoung et al., 2010), which is thought to be the center of cognitive control (Koechlin et al., 2003; Buschman and Miller, 2007). In addition, a longitudinal structural MRI study explored the relationship between cortical development and personality traits, and individuals with higher conscientiousness were associated with greater cortical thinning in the lateral and medial prefrontal cortices (Ferschmann et al., 2018), which further confirmed that conscientiousness is closely bonded with the prefrontal cortex. It can be speculated that conscientiousness may affect emotion regulation through the brain areas that are associated with cognitive control. However, it should be noted that emotion regulation has two different dimensions: cognitive reappraisal (CR) and expressive suppression (ES), which may be differently affected by conscientiousness.

CR is regarded as a cognitive strategy that interferes with emotional responses by modulating the meaning of the current situation (Buhle et al., 2014; Sheppes et al., 2014; Gross, 2015), which usually impacts the emotion-generative process in the early stage (Goldin et al., 2008). Unlike CR, ES is considered a subjective inhibition strategy that directly suppresses emotional responses (e.g., facial expressions and gestures) during the late stage of the emotion-generative process, which aims to directly control emotional behavior (Gross, 1998, 2015; Goldin et al., 2008; Joormann and Gotlib, 2010; Hermann et al., 2014). There are some similarities and differences between CR and ES in terms of brain structures and functions. CR involves the activation of widespread brain areas, such as the ventrolateral prefrontal cortex (vlPFC), dorsolateral prefrontal cortex (dlPFC) and dorsal anterior cingulate cortex (dACC; MacDonald et al., 2000; Ochsner et al., 2002; Phan et al., 2005), as well as decreases in the activity in some regions that are closely related to emotional experience, including the amygdala, posterior cingulate cortex and insula (Ochsner and Gross, 2005; Goldin et al., 2008). These findings suggest that CR is a cognitive strategy that is effective at down-regulating emotional experience and behavior (Goldin et al., 2008). The use of ES usually activates the prefrontal regions as well, especially the inferior frontal gyrus (IFG), dlPFC and dorsomedial prefrontal cortex (dmPFC; Goldin et al., 2008; Vanderhasselt et al., 2013). Although the PFC is thought to be a critical region for the two types of emotion regulation, there are differences in the temporal dynamics of the activation in the PFC between CR and ES, namely, CR activates the PFC in the early period, while ES activates the PFC in the late period (Goldin et al., 2008). In the study of brain structures, several studies have found positive relationships between ES and GMV in the anterior insula, dmPFC and ventromedial prefrontal cortex (vmPFC; Giuliani et al., 2011a; Kuehn et al., 2011; Hermann et al., 2014). CR has been found to be related to structural changes in the amygdala, dACC and lateral PFC (Giuliani et al., 2011b; Hermann et al., 2014; Vijayakumar et al., 2014); greater cortical thinning in the dlPFC and the vlPFC was significantly associated with CR scores (Vijayakumar et al., 2014). To some extent, these structural and functional studies have revealed that the dlPFC, vlPFC, amygdala and insula play a critical role in CR usage, while ES is associated with the vmPFC, dmPFC and IFG. Although there are some differences in the neural mechanisms of the two main modes of emotion regulation, the role of the PFC is equally important in CR and ES. Based on the above studies, considering the close bonds among conscientiousness, emotion regulation and the PFC, the lateral and medial PFC may act as mediators to affect the correlation between conscientiousness and the two types of emotion regulation. However, no study has directly investigated the role of the PFC in the correlation between conscientiousness and emotion regulation.

The aim of the current study was to investigate the relationship between conscientiousness and emotion regulation and to explore the brain structural basis of conscientiousness from the perspective of individual differences, as well as to examine whether the correlation between conscientiousness and emotion regulation may be mediated by specific areas associated with conscientiousness. First, we used the voxel-based morphometry (VBM) method to identify the association between conscientiousness and regional GMV at the whole-brain level. Second, we further examined which regions would be able to mediate the relationship between conscientiousness and emotion regulation (CR and ES). Given that some studies reported that conscientiousness reflects the abilities of inhibitory control and self-regulation (McCrae and John, 1992; DeYoung et al., 2010; Roberts et al., 2014), which are associated with structural variations in the dispersed frontal regions and the executive control network (DeYoung et al., 2010; Kapogiannis et al., 2013; Ferschmann et al., 2018), we hypothesized that the specific regional GMV related to conscientiousness would be involved in the dlPFC, medial PFC and IFG. In addition, considering that the executive control function plays a critical role in emotion regulation as well, we further hypothesized these regions related to conscientiousness would mediate the relationship between conscientiousness and emotion regulation.

## Materials and Methods

### Participants

A total of 351 (191 women and 160 men) individuals participated in this study as part of our ongoing project, which was designed to examine the associations between brain imaging, creativity, and mental health. All participants were right-handed and without a history of mental illnesses or neurological disorders. All participants provided written informed consent prior to the study. The Brain Imaging Center Institutional Review Board of Southwest China University approved this study and the experimental procedure.

### Assessment of Conscientiousness

The NEO-PI-R was used to measure the conscientiousness of each individual (Costa and McCrae, 1992). This scale is the most widely used measure of the FFM (five factor model) of personality structure, having high reliability (0.78; Yang et al., 1999).

### Assessment of Suppression

The Emotion Regulation Questionnaire (ERQ; Gross and John, 2003) was used to measure the emotion regulation ability of each individual. The ERQ is a self-report questionnaire that assesses two emotion-regulation strategies: CR (six items) and ES (four items). The scale consists of 10 items, and participants respond using a 7-point Likert scale (1 = strongly disagree, 7 = strongly agree). The test-retest reliability and a coefficient were 0.82 and 0.85 for reappraisal dimension, were 0.79 and 0.77 for suppression (Li et al., 2007).

### MRI Data Acquisition and Preprocessing

All the MRI data were acquired on a 3.0-T Siemens Trio MRI scanner (Siemens Medical, Erlangen, Germany). During data acquisition, the subjects were instructed to keep their heads still. Earplugs and foam padding were used to reduce the scanner noise and head motion, respectively. The high-resolution T1-weighted anatomical images were obtained with a magnetization-prepared rapid gradient echo (MPRAGE) sequence (repetition time [TR] = 1900 ms; echo time [TE] = 2.52 ms; inversion time = 900 ms; flip angle = 9°; resolution matrix = 256 × 256; field of view (FOV) = 256 × 256 mm^2^; slices = 176; thickness = 1.0 mm; and voxel size = 1 × 1 × 1 mm^3^).

The MRI scans were processed using SPM8 (Wellcome Department of Cognitive Neurology, London, UK^1^) through MATLAB 7.8 (Math Works Inc., Natick, MA, USA). Each MRI scan was first displayed in SPM8 to screen for any artifacts or gross anatomical abnormalities. For better registration, the orientation of the images was manually set to the anterior commissure. Then, the MRI scans were segmented into white matter, gray matter and cerebrospinal fluid using the “new segment” module in SPM8. Subsequently, we performed Diffeomorphic Anatomical Registration through Exponentiated Lie (DARTEL) algebra in SPM8 to create both a custom template and flow fields (that stored the deformation information) by iteratively aligning the gray matter images created in the previous step (Ashburner, 2007). The resulting custom template and flow fields were then used to create spatially normalized, smoothed (10 mm full-width at half-maximum Gaussian kernel) and resliced (with an isotropic voxel size of 1.5 mm) gray matter images. The reasons for using such a relatively large smoothing kernel were as follows: (a) smoothing can efficiently improve the signal-to-noise ratio; (b) since we used a large sample size here, it was foreseeable that the spatial registration (i.e., spatial normalization) would suffer from large individual anatomical variability, even with the optimized VBM methods (Ashburner, 2007); and (c) smoothing with a large kernel is known to reduce this misregistration effect.

### Statistical Analysis

Statistical analyses of the GMV data were performed using SPM8. We applied multiple linear regression to identify the brain region whose GMV was associated with individual differences in conscientiousness. We created a custom binary mask by including voxels with a gray matter value greater than 0.2 to avoid the partial volume effect. All subsequent statistical analyses were conducted in this mask. Gender, age and total GMV were included as nuisance covariates to remove any potential confounds. Within the custom mask, clusters with continuous suprathreshold voxels (*p* < 0.001) were initially identified. In the analyses of our studies, the p-maps were thresholded to yield an expected *p-value* of <0.05, with the FWE correction for multiple comparisons.

### Mediation Analysis

To test whether regional GMVs could explain the relationship between young adult conscientiousness and suppression ability, we performed a mediation analysis. Mediation analyses were conducted using the indirect macro designed for SPSS [45]. A mediating variable (M) is a variable that is part of the causal pathway by which an independent variable (X) affects a dependent variable (Y). In this study, X was the conscientiousness scores, Y was the suppression scores and M was the GMV of the regions associated with conscientiousness that were extracted from our own corrected results. This macro uses bootstrapped sampling to estimate the indirect mediation effect. In this analysis, 2000 bootstrapped samples were drawn and the bias-corrected, 95% bootstrap confidence intervals (CIs) were reported. CIs that did not include zero indicated a significant indirect effect of the independent variable on the dependent variable through the mediators (Preacher and Hayes, 2008).

## Results

### Descriptive Statistics

The demographic data and behavioral results are shown in Table 1. The mean conscientiousness score of the current sample was 161.72 (SD = 18.47). There was no significant difference between males and females in terms of conscientiousness scores (*p* > 0.05, *t* = 1.11, two-tailed *t*-test), which was consistent with the findings of previous studies.

**Table 1:** Demographic data.

	Males (*n* = 160)			Females (*n* = 191)		
Measure	Mean	SD	Range	Mean	SD	Range
Age	20.24	1.36	17–26	19.78	1.26	17–27
Conscientiousness	166.18	18.87	114–208	160.73	18.35	119–208
Suppression	3.93	6	6–27	3.70	5	5–26
Reappraisal	4.13	6	6–26	3.87	5	6–26

*Note: SD, standard deviation*.

### Correlations Between GMV and Conscientiousness Scores

**Figure 1:**
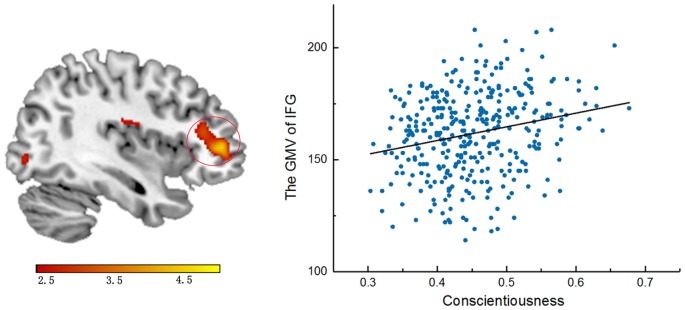
Anatomical correlations with conscientiousness scores.

After entering age, sex and global volumes of gray matter as covariates into the regression model, a multiple regression analysis revealed that the conscientiousness scores had significant, positive associations with the GMV in the right IFG (Left: MNI coordinates: 42, 42, 4.5; *t* = 4.85; cluster size = 615; *p* < 0.005), corrected by FWE at the cluster-level of *p* < 0.05 (see Figure 1).

### Mediation Results

**Figure 2:**
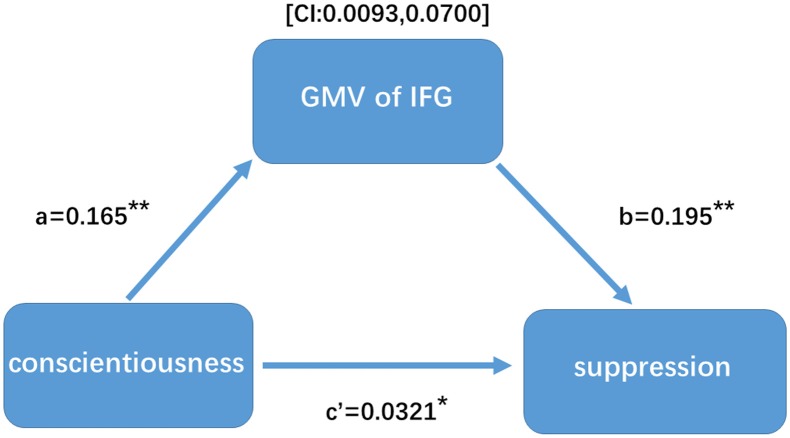
Gray matter volume (GMV) mediates the relationship between conscientiousness and expressive suppression.

Indirect mediation effects can be interpreted as the strength of the relationship between conscientiousness and suppression ability when accounting for mediating pathways. Conscientiousness was negatively associated with the GMV of the IFG (*r* = 0.231, *p* < 0.001). Suppression was negatively associated with the GMV of the IFG (*r* = 0.152, *p* < 0.01). Conscientiousness was positively associated with suppression (*r* = 0.173, *p* < 0.005). To test the significance of the indirect effects between conscientiousness and suppression, bootstrap resampling was used. The results showed a significant indirect effect (partial mediation effect) between conscientiousness and suppression, through the volume of the IFG (see Figure 2).

## Discussion

The present study investigated the associations among conscientiousness, emotion regulation and brain structure in a large, young sample. Conscientiousness was positively correlated with two types of emotion regulation strategies. The VBM results revealed that the GMV in the IFG was significantly and positively associated with conscientiousness, and the IFG acted as a mediator in the relationship between conscientiousness and ES.

Our results confirmed the close association between conscientiousness and emotion regulation, which was in line with previous research (Ivcevic and Brackett, 2014; Pocnet et al., 2017). Although some other studies indicated that there is a negative relationship between conscientiousness and ES, the difference between present and previous studies might cause by the different cultures, for example, chinese culture were more likely to encourage people to care about other people’s feelings instead of themselves, thus, people with high level of conscientiousness might more likely to suppress their own emotion and care about others. Combined with the finding that individuals with high conscientiousness are more likely to recover from negative stimuli and decreased negative affect (Javaras et al., 2012), we also provided direct evidence for the conscientiousness-emotion regulation association. From a theoretical standpoint, conscientiousness is mainly based on self-regulation or self-control (Eisenberg et al., 2014), and high levels of conscientiousness may be closely correlated with the attentional system, which usually influences the focus of emotional stimulus and helps to get rid of negative affect (Vollrath, 2001; Derryberry et al., 2003). These studies also provided evidence for the close relation between conscientiousness and emotion regulation.

The whole-brain VBM results revealed that conscientiousness was significantly and positively associated with GMV in a cluster that was mainly located in the right IFG, which was similar to the findings of a longitudinal study of brain structure development associated with personality traits (Ferschmann et al., 2018). As the key region of inhibitory control, the IFG has been implicated in inhibitory response paradigms (Swick et al., 2011; Bari and Robbins, 2013), especially in the go/no go task where the “no go” signal means to halt the response to the stimulus which directly leads to the activation of the right IFG (Aron et al., 2004). Individuals with high conscientiousness tend to be self-disciplined and organized in their work, and self-control or self-regulation is regarded as the core component of conscientiousness that contributes to academic and workplace performance (Higgins et al., 2007). The VBM results further confirmed the close association of conscientiousness with self-control or self-regulation from the perspective of structural variations. Meanwhile, although we failed to repeat the two other brain structural results related to the big five model of personality, the evidence is consistent in that conscientiousness is always closely linked to specific areas associated with self-control or self-regulation (DeYoung et al., 2010; Nostro et al., 2017).

More interestingly, the mediation analysis showed that the GMV in the right IFG partially mediated the correlation between conscientiousness and ES, rather than CR. CR is considered a cognitive-linguistic strategy that changes emotional responses by subjectively assigning a new meaning to the situation, and ES is a response-focused strategy that directly suppresses behaviors associated with emotional responses (Goldin et al., 2008; Vanderhasselt et al., 2013; Buhle et al., 2014; Sheppes et al., 2014; Gross, 2015). Compared with CR, ES may directly take up more inhibitory control resources and is associated with greater activation of the bilateral PFC and the IFG in response to emotional stimuli in the late stage of the emotion-generative process, and this strategy merely requires subjective inhibition of emotional responses (e.g., facial expressions and gestures) and may not require the involvement of other cognitive functions (Goldin et al., 2008; Vanderhasselt et al., 2013). In addition, taking into account the relationship between conscientiousness and ES embodied in the ability of inhibitory control (Caspi et al., 2005; Roberts et al., 2005, 2014; Goldin et al., 2008; Vanderhasselt et al., 2013; Eisenberg et al., 2014), the basis of the brain structure associated with inhibitory control can further explain the relationship between them. As expected, the IFG partially mediated the correlation between conscientiousness and ES. Furthermore, although there was a moderate correlation between conscientiousness and CR, no possible mediator was found from the perspective of brain structure. CR is not only involved in inhibitory control but also with more functions, such as working memory (Etkin et al., 2015). Specifically, CR aims to adjust emotional responses by altering the meaning of the stimulus rather than by directly suppressing (Gross and John, 2003; Gross, 2015); thus, conscientiousness may not affect CR through specific brain regions associated with inhibitory control, which contributed to the understanding the unique effect of the IFG in the two modes of emotion regulation. Taken together, the discussions highlight that the volumetric variations in the right IFG acted as a mediator in the correlation between conscientiousness and ES, based on the critical role of direct inhibitory control.

Our results are preliminary, and some limitations should be acknowledged. Despite the relatively large sample size, all participants of this study were young undergraduates, which may restrain the generalizability of these findings; thus, different sample groups need to be included to further validate these findings. In addition, the current results were correlational, and the mediation results also remained speculative, which suggests that the implementation of longitudinal studies may be required to further confirm the causal relationship between conscientiousness and emotion regulation and to consider other potential influencing factors. Of equal importance, task-based functional MRI research may be urgently needed to reveal the impact of individual differences in conscientiousness on the emotion regulation process. The study of conscientiousness and emotion regulation in some special fields can also be considered in the future, such as workplace impacts, legal consciousness and child development.

## Conclusion

In summary, the present study demonstrated that conscientiousness is correlated with emotion regulation, and the IFG volume mediates the relation between conscientiousness and ES rather than CR. These findings contributed by deepening the understanding of the relationship between conscientiousness and emotion regulation from the perspective of brain structural basis and highlighted the critical role of inhibitory control in these relationships.

## Author Contributions

CC and QJ designed the study. YM, JL and LH collected the data. CC and YM analyzed and interpreted the data; wrote the manuscript.

## Conflict of Interest Statement

The authors declare that the research was conducted in the absence of any commercial or financial relationships that could be construed as a potential conflict of interest.

## Footnotes

^1^www.fil.ion.ucl.ac.uk/spm
